# Residues of Legume AG41 Peptide Crucial to Its Bio-Insecticidal Activity

**DOI:** 10.3390/biom13030446

**Published:** 2023-02-27

**Authors:** Fatima Diya, Laurence Jouvensal, Isabelle Rahioui, Karine Loth, Catherine Sivignon, Lamis Karaki, Linda Kfoury, Francine Rizk, Pedro Da Silva

**Affiliations:** 1 INSA-Lyon, INRA, BF2I, UMR0203, Université de Lyon, 69621 Villeurbanne, France; 2 Plant Protection Department, Faculty of Agronomy, Lebanese University, Dekwaneh P.O. Box 146404, Lebanon; 3 Innovative Therapeutic Laboratory, Branch II, Department of Life and Earth Sciences, Faculty of Sciences, Lebanese University, Beirut P.O. Box 146404, Lebanon; 4 Centre de Biophysique Moléculaire, CNRS UPR 4301, 45071 Orléans, France; 5 UFR Sciences et Techniques, Université d’Orléans, 45071 Orléans, France; 6 INSA Lyon, INRAE, BF2I, UMR 203, Université de Lyon, 69621 Villeurbanne, France; 7 Department of Biological and Chemical Sciences, Lebanese International University, Beirut P.O. Box 146404, Lebanon

**Keywords:** peptide 3D structure, knottin peptide, bio-insecticidal peptide, structure function relationships, legume plant

## Abstract

Currently, crop protection relies heavily on chemical treatments, which ultimately leads to environmental contamination and pest resistance. Societal and public policy considerations urge the need for new eco-friendly solutions. In this perspective, biopesticides are effective alternatives to chemical insecticides for the control of various insect pests. Legumes contain numerous insecticidal proteins aimed at protecting their high nitrogen content from animal/insect predation. Investigating one such protein family at genome scale, we discovered a unique diversity of the albumin 1 family in the (model) barrel medic genome. Only some members retained very high insecticidal activity. We uncovered that AG41 peptide from the alfalfa roots displays an outstanding insecticidal activity against several pests such as aphids and weevils. Here we report the 3D structure and activity of AG41 peptide. Significant insights into the structural/functional relationships explained AG41 high insecticidal activity. Such observations pave the way for the development of bio-insecticides, with AG41 peptide as the lead compound.

## 1. Introduction

For centuries, pesticides have been commonly used for controlling weeds, pests and diseases leading to an average production loss of 50% [1]. Unfortunately, this has had serious impacts on water quality, biodiversity and human well-being. Endocrine-disrupting pesticides caused an incredible EUR 270 billion in health costs for Europe in 2017 [2]. Tang et al. [3] noted that 25,106 km^2^ of agricultural land worldwide is exposed to pesticide pollution, 31% of which is at high risk. Recently, the use of neonicotinoids—a class of pesticides that contributes to the loss of over 300,000 bee colonies per year—was banned [4]. In addition, the limited modes of action of current pesticides, combined with their injudicious and widespread use, have led to the rapidly resistant insect population selection [2,5].

**Figure 1:**
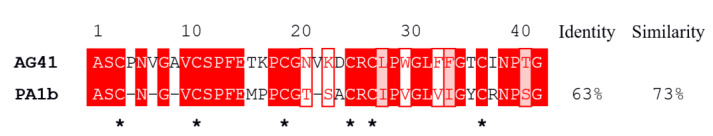
Alignment of PA1b and AG41 sequences. Identical residues are shown on a red background and similar residues are shown in red. The 6 cysteine residues are marked with black asterisks (*).

Hence, new strategies for controlling pests, of crops of economic importance, are required. New molecules with less impact on the environment have to be found. One of the most promising compounds is probably peptides, which are able to fight insects as well as fungal and bacterial attacks [6,7,8]. Investigating entomotoxic peptides within plants, particularly those consumed by mammals, can be a beneficial approach to develop sustainable and secure biopesticides for agriculture [8,9].One of the most promising of these compounds is the Albumin 1, subunit b peptide, extracted from leguminous plants. Legumes (Fabaceae) are important economic crops that provide food for humans, feed for livestock and raw materials for industry [10]. For instance, PA1b (Pea Albumin 1, subunit b) is a 37 amino acid knottin Disulfide-Rich Peptide (DRP) extracted from pea seeds (Figure 1) [11,12,13]. PA1b is an effective natural insecticide that can cause damage to many insects such as aphids, cereal weevils, mosquitos and moths [13,14]. It works by binding to the plasma membrane H^+^-ATPase (V-ATPase), specifically, on its subunits c and e in the insect midgut [12,15]. This proton pump plays an important role in the midgut of the insect, as it allows the absorption of nutrients [12,15]. The interaction of PA1b with V-ATPase triggers the apoptosis mechanism [16], resulting in insect death. Thus, V-ATPase proton pumps may be potential novel targets that have previously been widely ignored for use in insect control due to the lack of specificity of their chemical inhibitors. PA1b is the first specific peptidic V-ATPase inhibitor. A genomic exploration of PA1b homologues within *Medicago truncatula* legume identified AG41 (also known as *MtrA1013* isoform, encoded by the orphan TA24778 contig), a 41 amino acid peptide with six cysteines involved in three disulfide bonds (Figure 1), that displays a very high toxicity, almost ten times greater than that of the PA1b peptide [9,17].

In this study, we used NMR to solve the 3D structure of AG41 and compared it to that of PA1b. This structural analysis, together with the study of a rational collection of chemically synthesized alanine mutants of AG41 whose activities against Sf9 insect cells were assessed, allowed us to identify critical residues that enhance the activity of AG41. This approach provided significant insights into the functional elements responsible for the entomotoxic properties of AG41.

## 2. Materials and Methods

### 2.1. Synthesis and Purification of Peptides

The primary structure of the synthetic AG41 (ASCPNVGAVCSPFETKPCGNVKDCRCLPWGLFFGTCINPTG) is identical to the sequence of the 4291 Da (average molecular mass) isoform identified from the *Medicago truncatula* genome [9,17]. Reduced AG41 peptide and its reduced alanine mutant were chemically synthesized by the Genepep company (Saint-Jean-de-Védas, France). Then, the different peptides were folded according to the same optimized protocol reported for the production of the synthetic PA1b [18,19]. Briefly, they were buffered in Tris-HCl pH 8, 7 (final concentration 100 mM, containing 50% ethanol organic cosolvent, 1 mM EDTA, and reduced and oxidized glutathione in the following molar ratio: peptide/GSH/GSSG: 1/100/10 [18,19]. AG41 peptide and its alanine mutants were then purified by reverse-phase high-performance liquid chromatography (RP-HPLC). Peptide purities were evaluated by RP-HPLC and matrix-assisted laser desorption ionization-time of flight (MALDI-TOF) mass spectrometry. The peptide concentrations were determined using UV spectrophotometry at 280 nm (εTrp: 5500 M^−1^ cm^−1^). The concentration of the W29A variant was assessed by comparing its RP-HPLC peak area at 210 nm, against known quantities of pure standard peptides.

### 2.2. NMR Sample Preparation

AG41 was solubilized in a mixture of H_2_O:TFE-d3 (1:1 *v*/*v* ratio) at a concentration of 1.5 mM, and the solution pH was adjusted to 4.8 to achieve experimental conditions similar to those of PA1b.

### 2.3. NMR Experiments

2D 1H-NOESY, 2D 1H-TOCSY, 15N-HSQC and 13C-HSQC spectra (15N and 13C natural abundance) were recorded at 293 K on an Avance III HD BRUKER 700 MHz spectrometer prepared with a cryoprobe. 1H chemical shifts were related to the water signal (4.821 ppm at 293 K). NMR data were processed using Bruker’s Topspin 3.2TM and analyzed with CCPNMR (version 2.2.2) [20].

### 2.4. Structure Calculations

Structures were calculated using CNS [21,22] through the automatic assignment software ARIA2 (version 2.3) [23] with NOE-derived distances, hydrogen bonds (when the typical long or medium distance NOE cross peaks network characteristic of β-sheets had been observed—HN/HN, HN/Hα, Hα/Hα), backbone dihedral angle restraints (assessed with the DANGLE program [24], see Supplementary Table S1) and three disulfide bridges (Cys3-Cys24/Cys10-Cys26/Cys18-Cys36). The ARIA2 procedure used simulated annealing with torsion angle as well as Cartesian space dynamics with the default settings. The iterative protocol was completed until the assignment of the NOE cross peaks was achieved. The last run was performed with 1000 initial structures out of which 200 structures were refined in water. Ten structures were then selected on the basis of total energies and restraint violation statistics to represent the structure of AG41 in solution. The quality of the final structures was evaluated using PROCHECK-NMR [25] and PROMOTIF [26]. The figures were prepared with MOLMOL [27] and PYMOL [28].

### 2.5. Bioassays

To evaluate the affinity of synthetic AG41 and its mutants to PA1b receptor, ligand binding studies were conducted ^125^I-toxin [29]. Briefly, affinities (Ki) were measured competitively based on the level of specific binding and non-specific binding (non-specific binding typically accounts for 5–10% of total radioactivity; competitive experiments were not performed if non-specific binding was higher than 15% of total radioactivity, or if non-specific binding was greater than 60% of total binding). Total binding was assessed using 20 µg per assay of microsomal protein (sedimenting at 45,000 g) and 0.4 nM of I^125^-PA1b, while in the non-specific binding assays 1000 nM of unlabeled AG41 or alanine mutant peptide was added. The level of specific binding (i.e., total binding minus non-specific binding) was estimated based on three replicates. Competitive experiments were performed with 0.4 nM ^125^I-PA1b (specific radioactivity of approximately 1000 Ci.mmol^−1^) and competition was performed with AG41 and its alanine mutant peptides at concentrations ranging from 0.01 to 1000 nM.

Viability assays were performed on *Spodoptera frugiperda Sf9* insect cells in culture using the 3-(4,5-dimethylthiazol-2-yl)-2,5-diphenyltetrazolium bromide (MTT) protocol [30]. Briefly, *Sf*9 cells lines were grown at 27 °C in Lonza’s culture medium, supplemented with 5% fetal bovine serum (FBS) and 0.1% gentamicin. *Sf9* cells were seeded, in 96-well plates, 24 h prior to the experiments (15,000 cells/well) and were exposed to increasing concentrations of AG41 and its alanine mutant peptides for 24 h. Each concentration was performed in triplicate. Cell viability was determined by a colorimetric assay based on the ability of viable cells to reduce MTT. Cells were loaded with MTT solution (0.5 mg ml^−^^1^) and incubated at 27 °C for 2 h. Cell homogenates were used to measure absorbance at 550 nm using a microplate reader (MR 7000, Dynatech Laboratories Inc., Richmond, CA, USA).

### 2.6. Biological Data Analyses

The experimental Ki and LD5O data were assessed separately by non-linear regression analysis using the SIMFIT software package [31]. Biological results are expressed as mean ± S.E. (standard error).

## 3. Results and Discussion

### 3.1. The AG41 Peptide Fold Is Similar to That of PA1b

**Figure 2:**
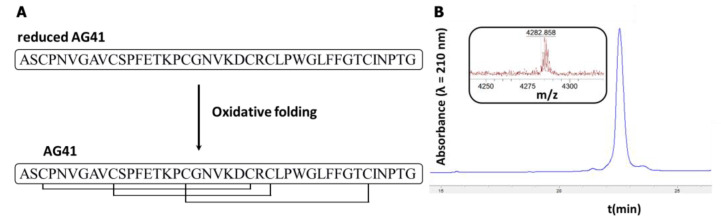
Chemical synthesis of AG41. (**A**) Schematic representation of the AG41 peptide oxidative folding. (**B**) RP-HPLC chromatogram and MALDI-TOF mass spectrum of the purified folded peptide.

The reduced AG41 peptide was engaged in the in vitro oxidative folding step following an optimized procedure, and the oxidized form was purified by RP-HPLC (Figure 2). The efficiency of the oxidative folding process for the AG41 peptide was approximately 60%, which is similar to that of the synthesized native PA1b [19]. The molecular mass of the AG41 peptide was measured by MALDI-TOF mass spectrometry (Figure 2b), and the measured mass was found to be consistent with the expected mass of the AG41 peptide containing three disulfide bridges [17].

The AG41 peptide was produced in sufficient amount to determine its 3D structure by NMR spectroscopy. The ^1^H NMR and ^15^N-HSQC spectra of the protein revealed a good dispersion of the amide chemical shifts, indicative of a well-folded peptide. Following the standard assignment strategy, the analysis of the 2D-TOCSY and NOESY spectra allowed almost complete assignment of the ^1^H chemical shifts (93.4%), except for residues Asn20 and Asp23 that could not be assigned, as well as Cys24 amide proton. The natural abundance heteronuclear NMR spectra provided assistance in unambiguously assigning the ^1^H chemical shifts, particularly in cluttered regions of the ^1^H TOCSY and NOESY spectra corresponding to side chains (BMRB ID 34742).

The 3D structures were calculated by taking into account a total of 920 NOE-derived distance restraints, added to 8 hydrogen bonds and 56 dihedral angles (Table 1, Table S1 and Figure S1). PA1b was shown to belong to the knottin structural group [32,33] based on NMR structural data [34], and the accuracy of its disulfide pairing was later confirmed by racemic crystallography [35]. Given that AG41 shares 73% similarity with PA1b (Figure 1), the same connectivity pattern was used for the structure calculation, namely Cys3-Cys24, Cys10-Cys26 and Cys18-Cys36.

Among the 200 water-refined structures of AG41, the 10 structures of lowest total energy compliance with all the experimental data and the standard covalent geometry were used for statistical analysis (Table 1). Restraints and coordinates were deposited as PDB entry 8AHK. Analysis of these 10 final structures with PROCHECK-NMR [25] showed that 96% of the residues are in the most favored or additionally allowed regions of the Ramachandran plot (Table 1).

**Figure 3:**
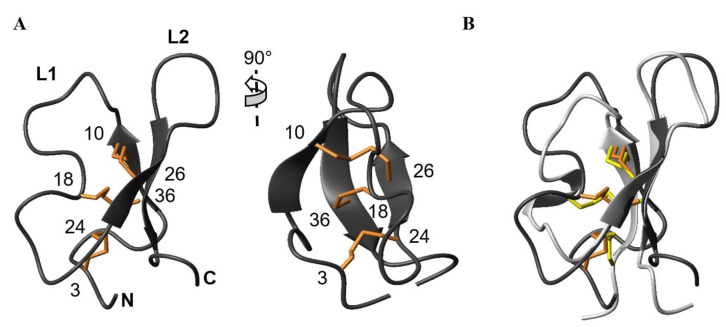
(**A**) Ribbon representation of AG41 lowest energy structure illustrating the inhibitory cysteine-knot fold with bridge 18–36 going through the macrocycle formed by disulfides 3–24 and 10–26 and the interconnecting backbone. (**B**) Superposition of ribbon representations of AG41 (dark grey, orange disulfide bridges) and PA1b (light grey, yellow disulfide connectivities) in the same orientation as in (**A**), fitted on the strands 2 and 3 that are involved in the β-hairpin.

AG41 displays the knottin fold typical of PA1b: a triple-stranded antiparallel β-sheet, with a topology of (+2x, −1), consisting of strand 1 (Ala8-Val9) hydrogen bonded to strand 3 (Thr35-Ile37), the latter being, in turn, hydrogen bonded to strand 2 (Arg25-Leu27) (Figure 3A). A long loop L_1_ (Cys10-Cys24) connects strand 1 to strand 2. This loop retains the CSPFE motif well-conserved among insecticidal PA1b homologues (11), of which the phenylalanine was proven to be a key residue for the entomotoxic activity [18]. A second loop, L_2_, interlinks strand 2 to strand 3, thus forming a 7:7 β-hairpin. The disulfide bridges of the cysteine-knot motif further stabilize the structure, connecting the two non-adjacent β-strands 1 and 2, on the one hand, and forcing loop L_1_ to fold back toward the central strand 3, on the other hand.

Not surprisingly given the high sequence similarity in this region, AG41 and PA1b hairpin loop backbones superimpose quite well (RMS deviation calculated over the β-strands residues 21–24,30–33 for PA1b/25–28,34–37 for AG41 = 0.37 ± 0.03 Å) (Figure 3b). A reverse γ-turn is observed between Leu27 and Trp29 in AG41, stabilized by the canonical C=O(27)—NH(29) hydrogen bond found on the 10 selected structures, as was observed between Ile23 and Val25 in PA1b. However, the following β-turns involving Gly30-Leu31-Phe32-Phe33 in AG41 and Gly26-Leu27-Val28-Ile29 in PA1b, respectively, adopt slightly different orientations, in line with the facing loop L_1_. The N-terminal parts of the two peptides adopt different conformations due to the presence of three additional residues between the first couple of cysteines (Cys3 and Cys10) in AG41, compared to PA1b (Figure 1). The structure being packed by the disulfides, incorporation of these extra residues is accommodated by a shortening of strand 1 in AG41 (Ala8-Val9) with respect to that in PA1b (Asn4-Gly5-Val6-Cys7), and by the formation of a type G1 β-bulge (Gly7, Ala8 opposite Cys36), is classically associated with a type II tight turn between Asn5 and Ala8. At the end of strand 1 and the beginning of loop L1, the VCSPFE pattern of AG41 on its own superimposes quite well with that of PA1b, whereas the second half of loop L_1_, between the third and fourth cysteines, does not. It is one-residue longer in AG41 (Cys18-Gly19-Asn20-Val21-Lys22-Asp23-Cys24) than in PA1b (Cys15-Gly16-Thr17-Ser18-Ala19Cys20). Furthermore, in AG41, loop L1 remains nearly flat, forming a plane with the β-hairpin strand 2, whereas in PA1b, a double-proline motif participates in a 3_10_ helix turn that curves loop L1 in a direction perpendicular to that of strand 2. Hence, the directions of the S-S bonds linking Cys15 and Cys32 in PA1b, and Cys18 and Cys36 in AG41, respectively, are not the same, whereas the other two disulfides superimpose roughly.

### 3.2. Structural Comparison Revealed the Potential Key Residues in the Activity of AG41

**Figure 4:**
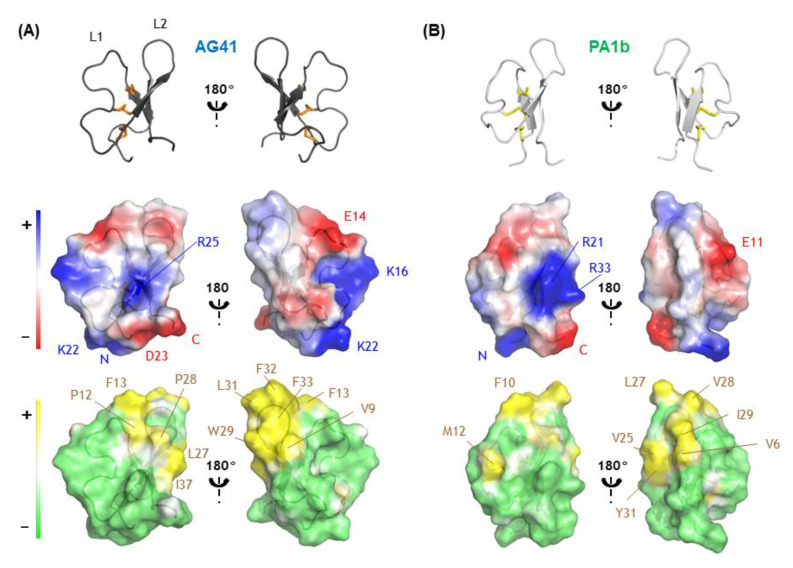
Electrostatic and lipophilic potentials calculated using the APBS plugin in PyMOL [28] and Platinum [36], respectively, at the molecular surfaces of (**A**) AG41, (**B**) PA1b. Rotating the left panel 180° around a vertical axis to match the right panel. Positive and negative areas as well as the residues defining these areas are shown in blue and red, respectively, while hydrophobic and hydrophilic areas are displayed in yellow and green, respectively. Residues involved in the definition of hydrophobic region are also indicated in brown.

To extend our comparison between AG41 and PA1b, we calculated the electrostatic and lipophilic potentials at the molecular surfaces of the proteins. Although both proteins share the same global charge (+1), AG41 counts three positively charged residues instead of two, and two negatively charged residues. Analysis of the electrostatic potentials at the surface of AG41 shows that the positive charges are more spread out at the surface of the molecule than in PA1b (Figure 4). They define two positive patches. The first one, rather extended, is formed by the N-terminus and the two lysine residues (Lys22 and Lys16). The second one surrounds Arg25 side chain and appears smaller and weaker than in PA1b due to the loss of the facing arginine on strand 3 of the β-sheet (in AG41, Ile37 replaces PA1b Arg33). However, Arg25 side chain still points out of the surface toward the solvent, identically to that of Arg21 in PA1b, which has been shown to play a pivotal role in the insecticidal activity of PA1b. Glu14 side chain, as Glu11 in PA1b, also points out on the opposite side of the molecule and defines a negative patch. The presence of a second negatively charged residue in the sequence of AG41, Asp23, does not affect the overall electrostatic profile of the molecule.

Analysis of the hydrophobic potentials at the surface of AG41 indicates that the molecule retains the amphipathic nature of PA1b. The hydrophobic pole is formed by the residues of the hydrophobic loop L_2_: Leu27, Pro28, Trp29, Leu 31, Phe32 and Phe33, and also by the facing residues Pro12 and Phe13 of L_1_. Unlike what is suggested by the homology model [17], AG41’s molecular surface does not present any groove between loop L_1_ and loop L_2_ at the top of this hydrophobic pole. In the AG41 sequence compared with that of PA1b (Figure 1), three aromatic residues replace aliphatic residues in loop L2, namely Trp29 instead of Val25, and Phe32 and Phe33 instead of Val28 and Ile29, respectively. Thus, AG41 appears even more hydrophobic so that it presents numerous bulky aromatic hydrophobic side chains in close proximity. This may be crucial for the interaction of AG41 with its V-ATPase target and explain the 10-fold gain in entomotoxic activity [17].

### 3.3. Synthesis and Structural Characterization of AG41 Mutants

Eleven single point alanine mutants were generated using the optimized synthesis procedure developed for the chemical production of AG41. This collection of mutants was selected on the basis of the structural analyses carried out earlier that had revealed the importance of these residues in defining the electrostatic and amphiphilic characteristics of AG41. Thus, the mutated residues (Table 2) were found in or around the L1 loop (Phe-13, Glu-14, Lys-16, Lys-22, Asp-23, Arg-25) and the L2 loop (Leu-27, Trp-29, Leu-31, Phe-32 and Phe-32). All the mutants were folded efficiently, as evidenced by RP-HPLC (Table S2), and the yield was similar to that of native AG41. The mutants were carefully identified using mass spectrometry. All of them have molecular masses corresponding to the theoretical masses of the mutants with the three disulfide bonds (Table S2). These findings clearly indicate that the cystine knot alone is sufficient to confer an extremely high degree of stability. Such evidences have already been reported for other cystine knot proteins, such as PA1b and its alanine mutants.

### 3.4. Bioactivity of Alanine Mutants of AG41

**Figure 5:**
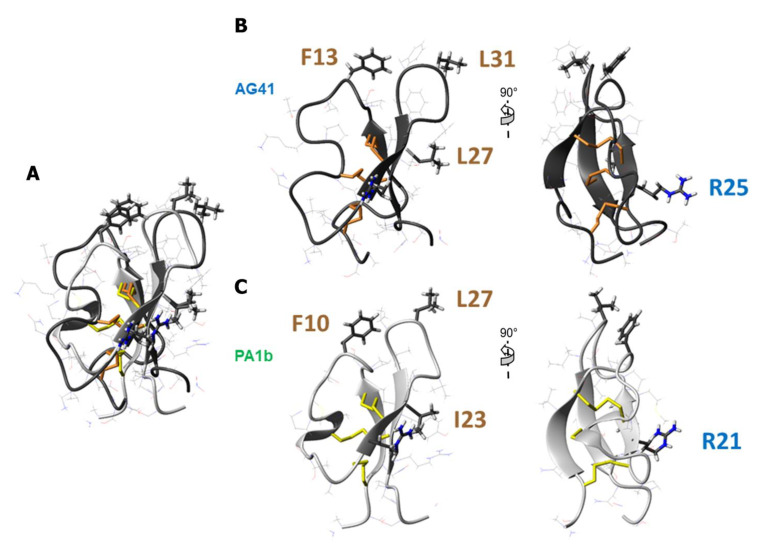
Key residues for PA1b and AG41 entomotoxicity. (**A**) Superposition of ribbon representations of AG41 (dark grey, orange disulfide bridges) and PA1b (light grey, yellow disulfide connectivities) with key residues represented in sticks, fitted on the strands 2 and 3 that are involved in the β-hairpin. (**B**) Ribbon representation of AG41 structure showing the key residues F13, R25, L27 and L31 for AG41 bioactivity in sticks. (**C**) Ribbon representation of PA1b structure displaying the key residues F10, R21, I23 and L27 for PA1b bioactivity in sticks.

The biological activities of the eleven mutants were assessed by measuring their affinities for the PA1b-binding site and their cellular toxicity on cultured Sf9 insect cells (LD_50_, lethal dose, 50%). Table 2 outlines the performance of the alanine mutants, showing that the replacement of a specific set of residues results in the lack of biological function. Indeed, substitution of Phe-13, Arg-25, Leu-27 and Leu-31 was observed to significantly reduce, or even abolish, the binding and toxic abilities of the peptide (Figure 5). These four residues of AG41 correspond to the residues Phe-10, Arg-21, Ile-23 and Leu-27 of PA1b, respectively (Figure 5). A former study on PA1b revealed their outstanding importance, both for binding and for toxicity [18]. The significance of these four residues for the insecticidal activity of AG41 is confirmed in this study. Mutation of Trp-29, Phe-32 and Phe33 residues, possibly responsible for the enhanced activity of AG41 with respect to PA1b, resulted in a decrease, but not a loss, of the binding and toxic abilities. These biological data, combined with the analysis of the hydrophobic potentials (Figure 4) clearly demonstrate the importance of the surface-exposed hydrophobic patch of AG41 as regards to its bioactivity. Moreover, mutation of Glu-14, Lys16 and Lys-22, which are not located around the four critical residues (Phe-13, Arg-25, Leu-27 and Leu-31) on the molecular surface, led to biological properties similar to those of the AG41 peptide. These results confirm that, with the exception of Arg-25, the charged residues are not primordial for the insecticidal activity of AG41, as previously shown for PA1b [18]. In this survey, we clearly identified the emerging crucial residue motif (Figure 5) that is linked to insecticidal function. This pattern was present in every insecticidal PA1b-like isoform identified so far in legumes. For example, the recent insecticidal PA1b-like peptide called α-astratide aM1 extracted from the roots of the legume *Astragalus membranaceus* exhibits the four-residue motif shown in Figure 5 [37]. This insecticidal residue fingerprint could be used to identify PA1b-like entomotoxic peptides in future sequenced legume genomes.

## 4. Conclusions

Through a rational production of AG41 mutants, we determined the amino acids essential for AG41 bioactivity (Phe-13, Arg-25, Leu-27 and Leu-31). Comparing the electrostatic and lipophilic potentials of AG41 to those of PA1b revealed the criticality of Trp-29, Phe-32 and Phe33 in its enhanced entomotoxic activity. These main hydrophobic residues likely play a significant role in both binding and cellular toxicity, raising an interesting prospect that the recognition of AG41 or PA1b with their V-ATPase target is essentially driven by hydrophobic interactions. This knowledge is a starting point to the creation of more powerful toxins.

## Figures and Tables

**Table 1:** NMR constraints and structural statistics.

NMR Restraints		
*Distance Restraints*		
Total NOE	920	
Unambiguous	845	
Ambiguous	75	
Hydrogen bonds	8	
***Dihedral Angle Restraints***	56	
***Covalent disulfide bridges***	Cys3-Cys24 Cys10-Cys26 Cys18-Cys36	
**Structural Statistics for the final 10 models of AG41**		
***Average number of violations per structure***		
NOEs ≥ 0.3 Å	0	
Hydrogen bonds ≥ 0.5 Å; ≥ 0.3 Å	0; 0.1	
Dihedrals ≥ 10°; ≥ 5°	0; 0.9	
***Average RMSD (pairwise, Å) ^a,b^***	bb (N-C_α_-C’)	All heavy atoms
Whole (2–41)	0.46 ± 0.09	0.92 ± 0.11
Triple-stranded β-sheet (8–9/25–27/35–37)	0.13 ± 0.04	0.62 ± 0.23
Loop L1 (10–24)	0.39 ± 0.13	1.02 ± 0.20
Hairpin loop L2 (28–34)	0.09 ± 0.04	0.67 ± 0,28
***Ramachandran Analysis***		
Most favored region and allowed region	96.0	
Generously allowed	4.0	
Disallowed	0.0	
***Energies* (kcal.mol ^−1^) *^a^***		
Electrostatic	−1313.83 ± 22.11	
Van der Walls	−306.91 ± 3.89	
Total energy	−1204.00 ± 17.30	
Residual NOE energy	26.83 ± 2.21	

^a^: values are provided as mean ± standard deviation (n = 10), ^b^: calculated using MOLMOL [27].

**Table 2:** Affinity to the PA1b receptor and insect cell toxicity of AG41 and its mutants. Values are provided as mean ± standard error.

AG41 and Its Mutants (Name and Sequence) ^a^		K_i_ ^b^ (nM)	LD_50_ ^c^ (nM)
AG41	ASCPNVGAVCSPFETKPCGNVKDCRCLPWGLFFGTCINPTG	1.3 ± 0.6	5.6 ± 1.8
F13A	ASCPNVGAVCSPAETKPCGNVKDCRCLPWGLFFGTCINPTG	-	-
E14A	ASCPNVGAVCSPFATKPCGNVKDCRCLPWGLFFGTCINPTG	1.2 ± 1.3	5.6 ± 1.8
K16A	ASCPNVGAVCSPFETAPCGNVKDCRCLPWGLFFGTCINPTG	3.6 ± 1.6	10.0 ± 4.0
K22A	ASCPNVGAVCSPFETKPCGNVADCRCLPWGLFFGTCINPTG	3.2 ± 1.7	14.2 ± 6.3
D23A	ASCPNVGAVCSPFETKPCGNVKACRCLPWGLFFGTCINPTG	1.2 ± 1.6	24 ± 9
R25A	ASCPNVGAVCSPFETKPCGNVKDCACLPWGLFFGTCINPTG	-	-
L27A	ASCPNVGAVCSPFETKPCGNVKDCRCAPWGLFFGTCINPTG	486 ± 11	907 ± 46
W29A	ASCPNVGAVCSPFETKPCGNVKDCRCLPAGLFFGTCINPTG	36.3 ± 6.7	89.0 ± 3.7
L31A	ASCPNVGAVCSPFETKPCGNVKDCRCLPWGAFFGTCINPTG	263 ± 8	-
F32A	ASCPNVGAVCSPFETKPCGNVKDCRCLPWGLAFGTCINPTG	39.8 ± 15.0	122 ± 12
F33A	ASCPNVGAVCSPFETKPCGNVKDCRCLPWGLFAGTCINPTG	15.0 ± 4.8	210 ± 8

(−) scores indicate a negative result (no toxicity nor binding in the tested toxin range). ^a^ Variant residue in AG41 mutants in comparison to AG41 sequence are boldfaced. ^b^ The K_i_ of AG41 and its mutants was evaluated using ^125^I-PA1b, according to [14]. ^c^ LD_50_ values were calculated from cultured Sf9 cell assays according to Rahioui et al. [30].

## Data Availability

All data are contained within the manuscript and Supplementary Materials.

## Supplementary Materials

The following supporting information can be downloaded at: https://www.mdpi.com/article/10.3390/biom13030446/s1. Table S1: Dihedral angles used during the structure calculation of AG41 and predicted by DANGLE, which predicts protein backbone dihedral angles and secondary structure assignments solely from amino acid sequence information, experimental chemical shifts and a database of known protein structures and their associated shifts; Table S2: Characterization of the oxidized form of AG41 and its mutants by RP-HPLC and MALD-TOF MS; Figure S1: Interaction Matrix of the NOE-derived restraints used during the structure calculation within all the residues.
