# Supplementary material for: Residues of Legume AG41 Peptide Crucial to Its Bio-Insecticidal Activity
Source: Biomolecules. 2023 Feb 27;13(3):446. doi: 10.3390/biom13030446 (PMC10046687; doi:10.3390/biom13030446)

SUPPLEMENTARY INFORMATION

Residues of legume AG41 peptide crucial to its bioinsecticidal activity

Fatima Diya[1,6,7, #], Laurence Jouvensal[2,3, #], Isabelle Rahioui[4], Karine Loth[2,3], Catherine Sivignon[4], Lamis Karaki[5], Linda Kfoury[6], Francine Rizk[7], Pedro Da Silva[1,*]

[1] Univ Lyon, INSA-Lyon, INRA, BF2I, UMR0203, F-69621, Villeurbanne, France [2] Centre de Biophysique Moléculaire, CNRS UPR 4301, Orléans, France.
[3] UFR Sciences et Techniques, Université d'Orléans, Orléans, F-45071, France
[4] Univ Lyon, INRAE, INSA Lyon, BF2I, UMR 203, F-69621, Villeurbanne, France
[5] Department of Biological and Chemical Sciences, Lebanese International University, Beirut, Lebanon
[5] Plant Protection Department, Faculty of Agronomy Lebanese University, Dekwaneh, Lebanon
[6] Plant Protection Department, Faculty of Agronomy Lebanese University, Dekwaneh, Lebanon
[7] Department of Life and Earth Sciences, Faculty of Sciences, Branch II, Innovative Therapeutic Laboratory, Lebanese University, Beirut, Lebanon
[#] Both authors contributed equally to this work *
Correspondence: pedro.da-silva@insa-lyon.fr (PdS)

This PDF file includes:

Table S1, Table S2 and Figure S1

Table S1: Dihedral angles used during the structure calculation of AG41 and predicted by DANGLE, which predicts protein backbone dihedral angles and secondary structure assignments solely from amino acid sequence information, experimental chemical shifts and a database of known protein structures and their associated shifts.

Residue Number	Residue Type	Phi (°)	Psi (°)	Phi Upper (°)	Phi Lower (°)	Psi Upper (°)	Psi Lower (°)	Experimental Chemical Shifts used for prediction
3	Cys	-76.50431	138.32407	-40.00000	-180.00000	-170.00000	50.00000	CA CB H HA N
4	Pro	-56.07213	-24.42870	-30.00000	-100.00000	10.00000	-60.00000	CA CB HA
6	Val	-55.12032	134.99919	-40.00000	-80.00000	160.00000	110.00000	CA CB H HA N
8	Ala	-72.54740	153.85650	-40.00000	-100.00000	179.99000	110.00000	CA CB H HA N
9	Val	-88.71359	133.78379	-60.00000	-150.00000	160.00000	100.00000	CB H HA N
10	Cys	-125.27354	152.27844	-90.00000	-170.00000	-170.00000	110.00000	CA CB H HA N
11	Ser	-123.78999	141.02900	-50.00000	-170.00000	179.99000	90.00000	CA CB H HA N
12	Pro	-57.59778	-23.01705	-40.00000	-90.00000	0	-50.00000	CA CB HA
13	Phe	-64.99299	-15.26339	-40.00000	-90.00000	0	-50.00000	CA CB H HA N
14	Glu	-64.42228	-	-30.00000	-90.00000	-	-	CA H HA N
15	Thr	-	-30.00729	-	-	0	-70.00000	CA CB H HA N
16	Lys	-94.22823	2.37205	-60.00000	-130.00000	40.00000	-30.00000	CA CB H HA N
17	Pro	-60.35738	138.92779	-40.00000	-90.00000	179.99000	110.00000	CA CB HA
18	Cys	64.98914	25.02203	90.00000	40.00000	50.00000	-10.00000	CB H HA N
20	Asn		147.34880			179.99000	80.00000	
21	Val	-90.18555	137.86279	-40.00000	-170.00000	179.99000	90.00000	CB H HA
23	Asp	55.00152	-	80.00000	40.00000	-	-	
24	Cys	-133.29604	158.09715	-100.00000	-160.00000	179.99000	120.00000	CA HA
25	Arg	-120.36707	137.11404	-80.00000	-170.00000	179.99000	100.00000	CA CB H HA N
26	Cys	-106.02843	120.52099	-70.00000	-150.00000	160.00000	90.00000	CA CB H HA N
27	Leu	-130.27225	148.40190	-60.00000	-180.00000	179.99000	100.00000	CB H HA N
28	Pro	-64.96722	-	-40.00000	-90.00000	-	-	CA CB HA
29	Trp	-60.46409	138.26668	-40.00000	-100.00000	179.99000	110.00000	CA CB H HA N
31	Leu	-77.68877	-11.64527	-40.00000	-120.00000	40.00000	-40.00000	CB H HA N
32	Phe	-79.43258	-19.91530	-40.00000	-120.00000	30.00000	-60.00000	CA CB H HA N
33	Phe	-90.54127	-	-60.00000	-140.00000	-	-	CA CB H HA N
35	Thr	-128.71653	141.03175	-70.00000	-170.00000	170.00000	100.00000	CA CB H HA N
36	Cys	-100.12062	138.34309	-40.00000	-180.00000	-170.00000	90.00000	CA CB H HA N
37	Ile	-131.07080	156.16955	-100.00000	-170.00000	-170.00000	120.00000	CA CB H HA N
38	Asn	-80.74117	154.93556	-40.00000	-110.00000	-170.00000	100.00000	CA CB H HA N
39	Pro	-57.64849	143.24565	-40.00000	-90.00000	179.99000	120.00000	CA CB HA

Table S2: Characterisation of the oxidized form of AG41 and its mutants by RP-HPLC and MALD-TOF MS

Peptides	Retention time[a] (min)	monoisotopic observed mass[b] $[M+H]^+$	Calculated for	monoisotopic theoretical mass $[M+H]^+$
AG41	22.62	4282.86	$C_{187}H_{280}N_{50}O_{54}S_6$	4282.90
F13A	20.82	4206.59	$C_{181}H_{276}N_{50}O_{54}S_6$	4206.87
E14A	21.98	4224.61	$C_{185}H_{278}N_{50}O_{52}S_6$	4224.90
K16A	23.34	4225.97	$C_{184}H_{273}N_{49}O_{54}S_6$	4225.84
K22A	23.31	4225.57	$C_{184}H_{273}N_{49}O_{54}S_6$	4225.84
D23A	22.88	4238.98	$C_{186}H_{280}N_{50}O_{52}S_6$	4238.91
R25A	23.75	4198.21	$C_{184}H_{273}N_{47}O_{54}S_6$	4197.84
L27A	21.54	4240.95	$C_{184}H_{274}N_{50}O_{54}S_6$	4240.86
W29A	19.09	4168.64	$C_{179}H_{275}N_{49}O_{54}S_6$	4167.86
L31A	19.81	4240.83	$C_{184}H_{274}N_{50}O_{54}S_6$	4240.86
F32A	20.75	4207.12	$C_{181}H_{276}N_{50}O_{54}S_6$	4206.87
F33A	19.86	4207.02	$C_{181}H_{276}N_{50}O_{54}S_6$	4206.87

[a]: The peptides were eluted on a C18 reversed-phase column with a linear gradient of MeCN/H_2O/0.04%TFA (20%60% in MeCN over 30 min) at a flow rate of 1mL/min. [b]: matrix used: □-cyano-4-hydroxy-cinnamic acid (HCCA), co-crystallized using the dry droplet method

Figure S1: Interaction Matrix of the NOE derived restraints used during the structure calculation within all the residues.

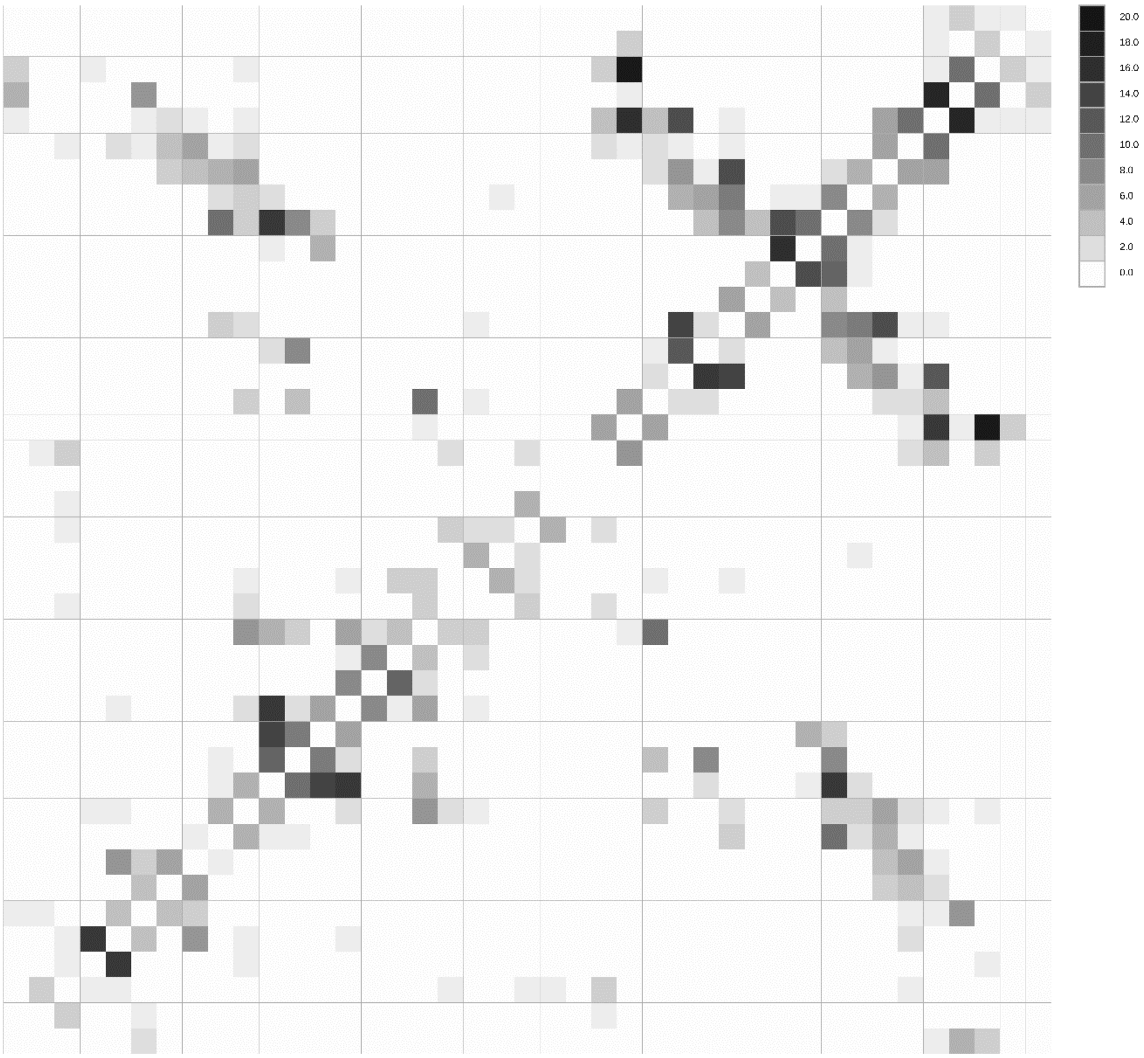

Supplement: Supplementary file 1 [file biomolecules-13-00446-s001.zip › biomolecules-2241311-supplementary.pdf]
